# Continuous-time robust frequency regulation in isolated microgrids with decentralized fixed structure μ-synthesis and comparative analysis with PID and FOPID controllers

**DOI:** 10.1038/s41598-024-70405-7

**Published:** 2024-09-05

**Authors:** Abdallah Mohammed, Ahmed Kadry, Maged Abo-Adma, Adel El Samahy, Rasha Elazab

**Affiliations:** Faculty of Engineering, Helwan University, Cairo, Egypt

**Keywords:** Microgrid, Frequency regulation, Decentralized controller, Fixed structure μ-synthesis, Robust frequency control, Energy science and technology, Engineering

## Abstract

Isolated microgrids, which are crucial for supplying electricity to remote areas using local energy sources, have garnered increased attention due to the escalating integration of renewable energy sources in modern microgrids. This integration poses technical challenges, notably in mitigating frequency deviations caused by non-dispatchable renewables, which threaten overall system stability. Therefore, this paper introduces decentralized fixed structure robust μ-synthesis controllers for continuous-time applications, surpassing the limitations of conventional centralized controllers. Motivated by the increasing importance of microgrids, this work contributes to the vital area of frequency regulation. The research challenge involves developing a controller that not only addresses the identified technical issues but also surpasses the limitations of conventional centralized controllers. In contrast to their centralized counterparts, the proposed decentralized controllers prove more reliable, demonstrating enhanced disturbance rejection capabilities amidst substantial uncertainties, represented through normalized co-prime factorization. The proposed controllers are designed using the D-K iteration technique, incorporating performance weight filters on control actions to maintain low control sensitivity and ensure specific frequency band operation for each sub-system. Importantly, the design considers unstructured uncertainty up to 40%, addressing real-world uncertainties comprehensively. Rigorous robust stability and performance tests underscore the controller's superiority, demonstrating its robustness against elevated uncertainty levels. Robust stability is verified for all controllers, with the proposed controller showing robust stability against up to 171% of the modeled uncertainty. Notably, the controller boasts a fixed structure with lower order compared to other H-infinity controllers, enhancing its practical implementation. Comparative analyses against Coronavirus Herd Immunity Optimizer tuned Proportional-Integral-Derivative (CHIO-PID) controller and CHIO tuned Fractional-Order Proportional-Integral-Derivative (CHIO-FOPID) controller further validate the superior performance of the proposed solution, offering a significant step towards ensuring the stability and reliability of microgrid systems in the face of evolving energy landscapes.

## Introduction

Recently, isolated microgrids have become a focal point for researchers due to their potential to fulfill the energy needs of remote areas. Despite the attractiveness of renewable energy sources in modern microgrids, the escalating integration of renewables introduces technical challenges, including low inertia and output power fluctuations resulting from the stochastic behavior of these sources. Such challenges lead to high frequency deviations and voltage instabilities^1^. These issues can be mitigated by incorporating traditional energy sources such as diesel generators, batteries, flywheels, superconducting magnetic energy storage systems, and ultra-capacitors^2–4^. However, each energy storage system has its drawbacks, with energy storage systems suffering from high frequency response and maintenance problems, flywheels and superconducting magnetic energy storage systems facing energy density issues, and ultra-capacitors standing out with fast power response^5^. Microgrids often employ both centralized and decentralized control systems^6^. While centralized control is straightforward, it faces reliability issues, as any interruption in the central controller affects the system's stability, and expanding or scaling this form of control is challenging. In contrast, decentralized control, with separate and adaptable local controllers, offers a more dependable, flexible, and extendable solution^7^.

Droop control has emerged as a decentralized technique with a key advantage—it does not rely on communication but instead focuses on regional measurements. However, droop control approaches tend to overlook the dynamics of generators, impacting the control response and performance. To guarantee dynamic stability in the presence of a high level of disturbance, consideration and analysis of generator dynamics become imperative^8^.

Existing literature explores various control strategies such as model predictive control (MPC)^9^ and modified model predictive control (MMPC)^10^. However, these approaches often face limitations due to challenges in practical implementation. While MPC and MMPC can offer improved performance in theory, their practical application is hindered by computational complexity and the need for precise modeling, which can be difficult to achieve in real-world scenarios^11^.

Additionally, Proportional-Integral-Derivative (PID) controllers^12,13^ and Fractional Order PID (FOPID) controllers^14,15^ are widely used in practice due to their simplicity and effectiveness in many applications. However, these controllers also have limitations. PID controllers can struggle with system uncertainties and non-linearities^16^, leading to suboptimal performance under certain conditions. FOPID controllers, while offering better tuning flexibility and robustness than traditional PID controllers, still face challenges related to parameter tuning and implementation complexity^17^.

Thus, while these control strategies are extensively employed in practice, their limitations, including system uncertainties, lack of a systematic framework, and practical implementation challenges, must be acknowledged and addressed to enhance their effectiveness in ensuring dynamic stability and optimal performance.

In^18,19^, the H∞ control method was illustrated as a centralized controller. However, system uncertainties were not addressed. The decentralized H∞ loop shaping controllers for frequency regulation in the microgrid are presented in^16^. However, each controller was shaped for each generation unit separately; therefore, interconnections between distributed generation units were not taken into consideration. Furthermore, they did not have a framework for loop shaping that was systematic. Additionally, it is difficult to implement the designed controller because the order of the designed H∞ controller is equal to the order of the plant. The designed controller should have a fixed-structure controller, satisfy robust stability and performance requirements, and have an acceptable order to be implemented. Sliding mode controllers^20^, although robust, can suffer from chattering issues, which can be detrimental to system components^21^. Table 1 provides a comparison between the proposed μ-synthesis controller and the existing controllers, demonstrating that the proposed μ-synthesis controller addresses all the challenges present in existing control systems.

**Table 1:** Comparison between the proposed μ-synthesis controller and existing controllers.

Controller	System uncertainty modeling	Controller order	Weight filter
μ-synthesis robust controller	Deal with a high level of system uncertainties	Second order controller	Weight filters are taken into considerations to operate each sub-system within a specific bandwidth
Robust controllers (H∞ loop shaping and Sliding mode controllers)	Deal with a high level of system uncertainties	The controller order equal to the system order	Weight filters are not taken into considerations
Conventional Controllers (drop control, model predictive control, PID, and FOPID)	Suffer from limitations due to system uncertainties	The controller order may be lower order than the system order	Weight filters are not taken into considerations

In this paper, a continuous-time μ-synthesis robustness decentralized controller is proposed to address the frequency deviation challenges in isolated microgrids. This technique was chosen due to its superior ability to handle system uncertainties and ensure robust performance across various operating conditions. Unlike traditional controllers, μ-synthesis provides a systematic framework for designing controllers that can explicitly consider and compensate for model uncertainties and external disturbances. This is particularly crucial in isolated microgrids where uncertainties can significantly impact system stability and performance. Furthermore, μ-synthesis allows for the inclusion of weight filters, which can be tailored to operate each subsystem within a specified bandwidth frequency, thereby optimizing the performance of the entire microgrid.

In summary, the research gap addressed by this paper is the need for a decentralized control strategy that can effectively manage frequency deviations in isolated microgrids while considering practical implementation challenges such as controller order and weight filter design. By leveraging the robustness and systematic design framework of μ-synthesis, this paper contributes a viable solution to enhance the stability and performance of isolated microgrids, outperforming other control strategies that struggle with system uncertainties and practical implementation issues.

This designed controllers specifically tackle unstructured uncertainties, such as operating point uncertainty and fluctuations in the output power of renewable energy sources, represented using normalized co-prime factorization. Performance weight filters are applied to control actions to maintain low control sensitivity and ensure optimal operation within specific frequency bands for each sub-system. Through rigorous robust stability and performance tests, the superior capability of the proposed controller to withstand a broad range of uncertainties is demonstrated. Comparative analysis with CHIO-PID and CHIO-FOPID controllers, considering input disturbances and diverse system uncertainties, underscores the practical advantages of this proposed controller, with a simplified order enhancing its feasibility.

This work builds upon recent studies, contributing a comprehensive approach that not only embraces low-order implementation but also considers uncertainties and integrates weight filters for improved control sensitivity and system performance.

Recent developments in the field include studies on decentralized low-order μ-synthesis controllers in^22^, which, although practically implemented with a low order, did not delve into the consideration of weight filters. Additionally, investigations in^23,24^ addressed μ-synthesis controllers in decentralized settings but focused primarily on system order considerations rather than practical implementation aspects. Another noteworthy study proposed an eighth-order μ-synthesis controller in^25^ with only 20% structure uncertainty, emphasizing the need for a lower-order controller for practical implementation. Other studies in^26,27^ suggested H∞ controllers but with the same system order and without weight filter consideration.

This work stands out by introducing decentralized second-order μ-synthesis controllers for frequency regulation in isolated microgrids. The proposed controller is designed with practicality in mind, featuring a low order that eases implementation. It effectively handles 40% unstructured uncertainties, accounting for unpredictable variations in all parameters—a significant advantage over structured uncertainties. This approach not only acknowledges and addresses the challenges posed by random fluctuations but also incorporates weight filters to enhance overall performance. This approach addresses critical issues, ensuring robust stability and effective disturbance rejection in the face of significant uncertainties. This study’s comparative analysis positions it as a significant advancement in the quest for reliable and practical frequency regulation in isolated microgrids. This paper is structured into sections that cover microgrid modeling, uncertainty modeling, the proposed controller's design process, simulation results, and concluding remarks.

## Microgrid modeling

**Figure 1:**
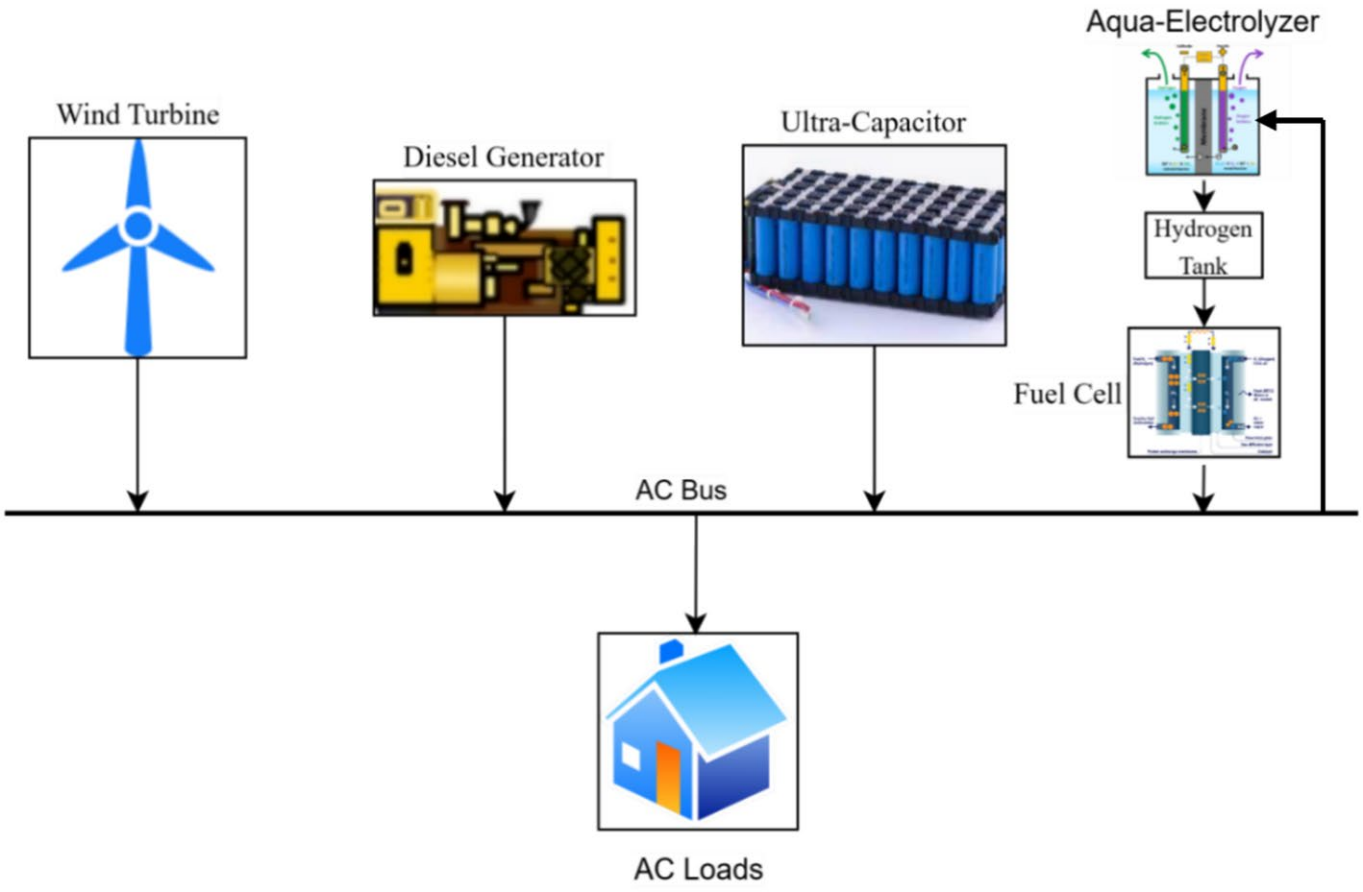
Microgrid configuration.

Figure 1 depicts the configured architecture of an isolated hybrid microgrid under examination. The microgrid ensemble encompasses a suite of energy sources, including a diesel generator, fuel cell, electrolyzer, wind generation system, and an ultra-capacitor serving as an energy storage system^28,29^. The diesel generator is supplied with a speed governor, which functions to regulate the speed of the diesel engine. Concurrently, a blade pitch control mechanism is employed within the wind turbine system to ensure that the speed of the wind turbine generator remains within prescribed operating limits, preventing it from surpassing the designated maximum power set point amidst fluctuating wind speeds^29^.

### Wind power modeling

The wind turbine generator's output power is proportional to the cube of the wind speed ${V}_{wind}$. The mechanical power output of a wind turbine can be defined in Eq. (1)^27^:

1 $$ P_{{WTG}}  = \left( {{\frac{1}{2}}\ast \rho _{a} \ast C_{p} \ast A_{b} \ast V_{{wind}} } \right) $$

${{\text{A}}}_{{\text{b}}}$: blade area $({\text{m}}^{2})$; ${\rho }_{a}:$ air density (kg/m$^{3}$); and ${C}_{p}$: power co-efficient which is a function of tip speed ratio $(\lambda )$ and blade pitch angle $(\beta )$.

According to Eq. (1), the intermittent characteristics inherent in a renewable wind generation system can influence the power quality and overall performance of a hybrid microgrid. To mitigate potential disruptions, the blade pitch control methodology is implemented to minimize errors in power generation and mitigate frequency variations. The continuous monitoring of wind turbine speed is facilitated by the blade pitch control mechanism, which actively engages within the turbine's feedback control system, ensuring precise and responsive adjustments.

### Fuel cell modeling

Fuel cells serve as static energy converters, transforming the chemical energy of fuel into electrical energy through electrochemical reactions. Due to their remarkable performance, minimal environmental footprint, and versatile modular structure, fuel cells are pivotal assets in hybrid distributed generation power systems. Compared with a fuel tank and an inverter for the conversion of DC power to AC power, the fuel cell system, despite its inherent nonlinear model and high-order characteristics, is effectively represented by a three-order model for frequency studies^13,28,29^. The model is given in Eq. (2)^31^ in terms of the inverter, interconnection, and fuel cell time constants ${T}_{IN}$, ${T}_{IC}$, and ${T}_{FC}$.

2 $$FC(S)=\frac{{K}_{FC}}{(1+{T}_{IC}s)(1+{T}_{IN}s)(1+{T}_{FC}s)}$$

### Aqua-electrolyzer modeling

Aqua-electrolyzer generate hydrogen, which is required by fuel cells by utilizing renewable energy as an electricity source. The aqua-electrolyzer model is defined in Eq. (3) in terms of a time constant ${T}_{AE}$ and a gain ${K}_{AE}$.

3 $${G}_{AE}(S)=\frac{{K}_{AE}}{(1+{T}_{AE}s)}$$

### Ultra-capacitor modeling

Ultra-capacitors are capacitors with a high capacity and a low voltage rating of approximately 2.5 V. They are used to store extra power generation in microgrids and then deliver it in a short period of time at peak-load demand^13^. Equation (4) expresses the ultra-capacitor's transfer function as a function of a time constant ${T}_{UC}$ and a gain ${K}_{UC}$.

4 $${G}_{UC}(S)=\frac{{K}_{UC}}{(1+{T}_{UC}s)}$$

In practice, several ultra-capacitors are connected together to achieve the required terminal voltage and energy storage capacity.

### Diesel generator modeling

In response to the frequency deviation signal, the frequency controller of the distributed diesel generating unit initiates a control action directed towards the speed-gear changer of the diesel engine. Employing the frequency variation within the power system as a feedback input, this mechanism ensures the stability of the system. Striking a balance is crucial, as rapid operation of the speed-gear changer may lead to increased wear and tear on the engine, while sluggish operation may compromise overall system performance. Hence, the presence of an effective frequency controller is imperative for seamless operation of the system.

**Figure 2:**
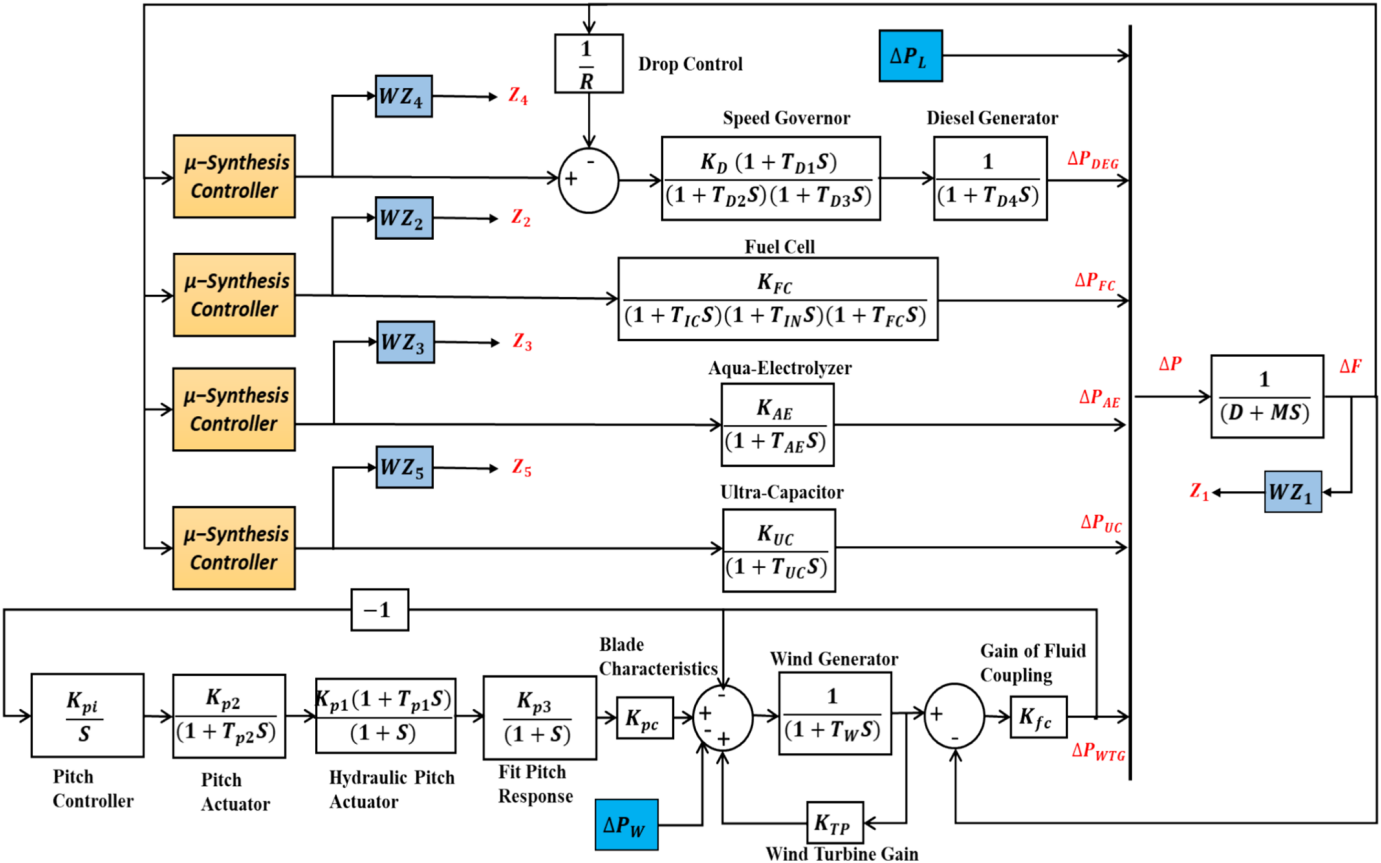
Overall block diagram for the studied system.

A comprehensive block diagram of the investigated system is shown in Fig. 2. This system encompasses diverse components, including a diesel generator, an electrolyzer, an ultra-capacitor serving as an energy storage element, a fuel cell, and a wind generation system. The intricacy lies in orchestrating the operation of each generation unit within specified frequency bands, accounting for the unique dynamics inherent to each unit, as elucidated in subsequent sections. Table 2 provides a detailed overview of the parameters associated with each distributed generation unit within the examined system.

**Table 2:** The parameters of the studied hybrid microgrid.

Parameter	Value	Nomenclature
${K}_{FC}$	0.01	Gain constant of the fuel cell
${K}_{IC}$	1	Gain constant of interconnection
${K}_{IN}$	1	Gain constant of inverter
${T}_{IC}$	0.004	Time constant of the interconnection
${T}_{IN}$	0.04	Time constant of the inverter
${T}_{FC}$	4	Time constant of the fuel cell
${K}_{AE}$	0.04	Gain constant of the aqua-electrolyzer
${K}_{UC}$	-0.7	Gain constant of the ultra-capacitor
${{\text{T}}}_{{\text{AE}}}$	0.002	Time constant of the aqua-electrolyzer
${{\text{T}}}_{{\text{UC}}}$	0.9	Time constant of the ultra-capacitor
${{\text{K}}}_{{\text{fc}}}$	1.5	Fluid coupling
${{\text{K}}}_{\text{p}2}$	1.25	Hydraulic actuator gain
${\text{K}}_{\text{p}3}$	1.4	Gain of fit pitch response
${\text{T}}_{\text{p}1}$	0.6	Time constant of the pitch control
${\text{T}}_{\text{p}2}$	0.04	Time constant of the hydraulic pitch actuator
${\text{K}}_{\text{pc}}$	0.08	Blade characteristic constant
${\text{K}}_{\text{pi}}$	6.1	Integral controller gain of pitch controller
${\text{K}}_{\text{D}}$	0.4	Speed governor gain
M	0.4	Inertia constant
D	0.03	Damping constant
R	5	Drop constant

Diesel generator units (2 units, 150 kW each), wind system (2 units, 150 kW each), fuel cell (4 units, 50 kW each), ultra-capacitor system = 200 kW, load demand = 600 kW.

## Uncertainty modeling

**Figure 3:**
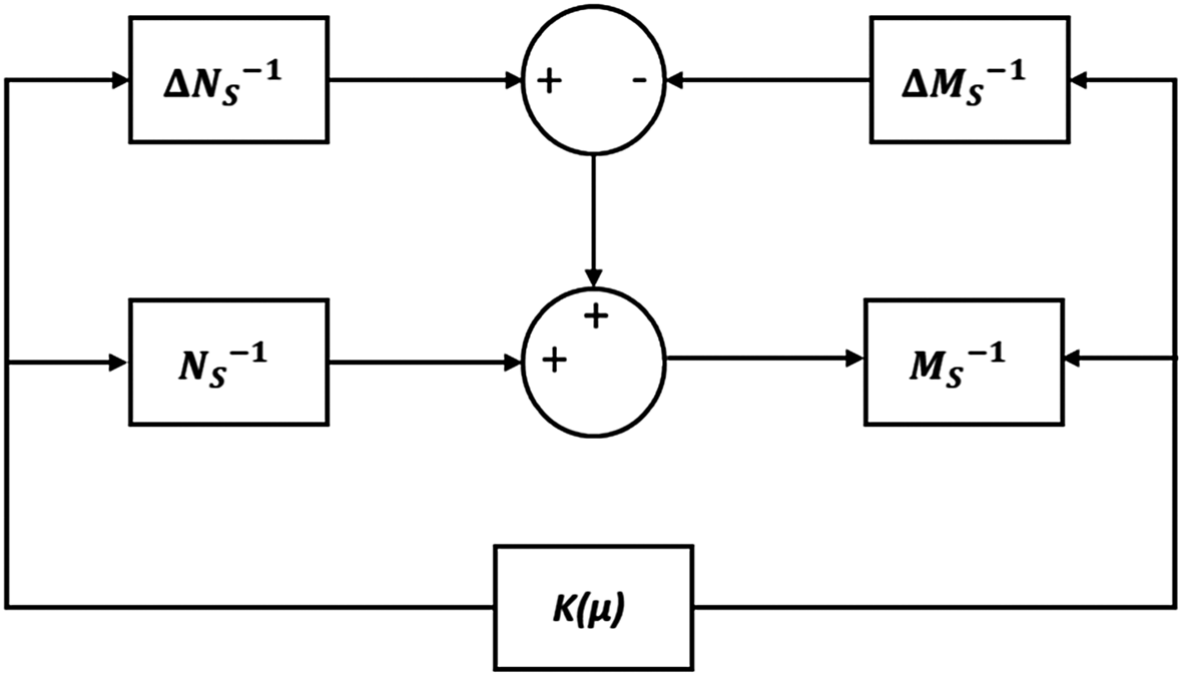
Plant with uncertainty model and µ-synthesis controller.

System uncertainties can arise from the variability of model parameters or the inherently fluctuating characteristics of renewable energy sources. To address these intricate uncertainties, this paper incorporates the normalized co-prime factorization technique^32^ as a robust modeling approach. The standardized normalized co-prime factor is defined in Eq. (5) and the perturbed plant ${P}_{\Delta }$ is given by Eq. (6).

5 $${P}_{l}= {M}_{S}^{-1} {N}_{S}$$

6 $${P}_{\Delta }=\left\{\begin{array}{c}{({M}_{S}+\Delta {M}_{S})}^{-1} ({N}_{S}+ \Delta {N}_{S})\\ \Vert \Delta {M}_{S}\Delta {N}_{S}\Vert \le \frac{1}{\gamma }\end{array}\right.$$

where Δ${M}_{S}$ and Δ${N}_{S}$ are transfer functions that simulate the plant's unstructured uncertainty as shown in Fig. 3.

**Figure 4:**
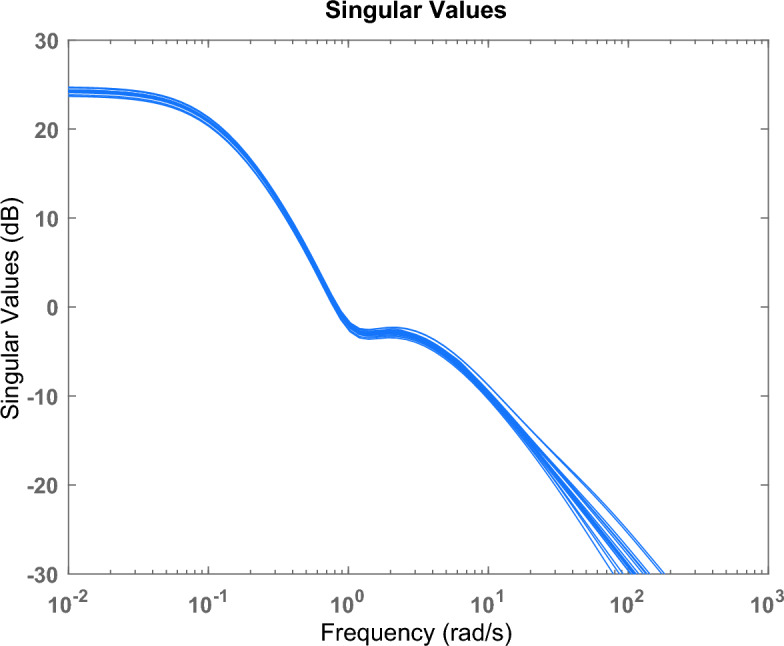
Frequency response for the studied microgrid with the uncertainty model.

Figure 4 shows the frequency response of the studied microgrid with the uncertainty model (40% unstructured uncertainty).

## Proposed controllers

In this section, a μ-synthesis robust decentralized controller, CHIO-PID, and CHIO-FOPID decentralized controllers are discussed.

### μ-synthesis robust controller

One of the most important issues in control theory that has long been studied is robustness. A key measure of a control system's robustness is how sensitive it is to both external and internal disturbances. Several robust strategies have been introduced in ^33–35^ to guarantee robust stability and performance under the influence of high levels of uncertainty.

In this paper, a μ-synthesis robust decentralized controller is designed to control the isolated microgrid frequency. The designed control addresses system unstructured uncertainties such as operating point uncertainty and fluctuations in the output power of renewable energy sources. For complicated systems with any type of uncertainty, the µ-synthesis robust control enables to design of a multivariable optimal robust controller. It extends the H∞ synthesis and minimizes the closed loop gain of the system to find a robust controller.

**Figure 5:**
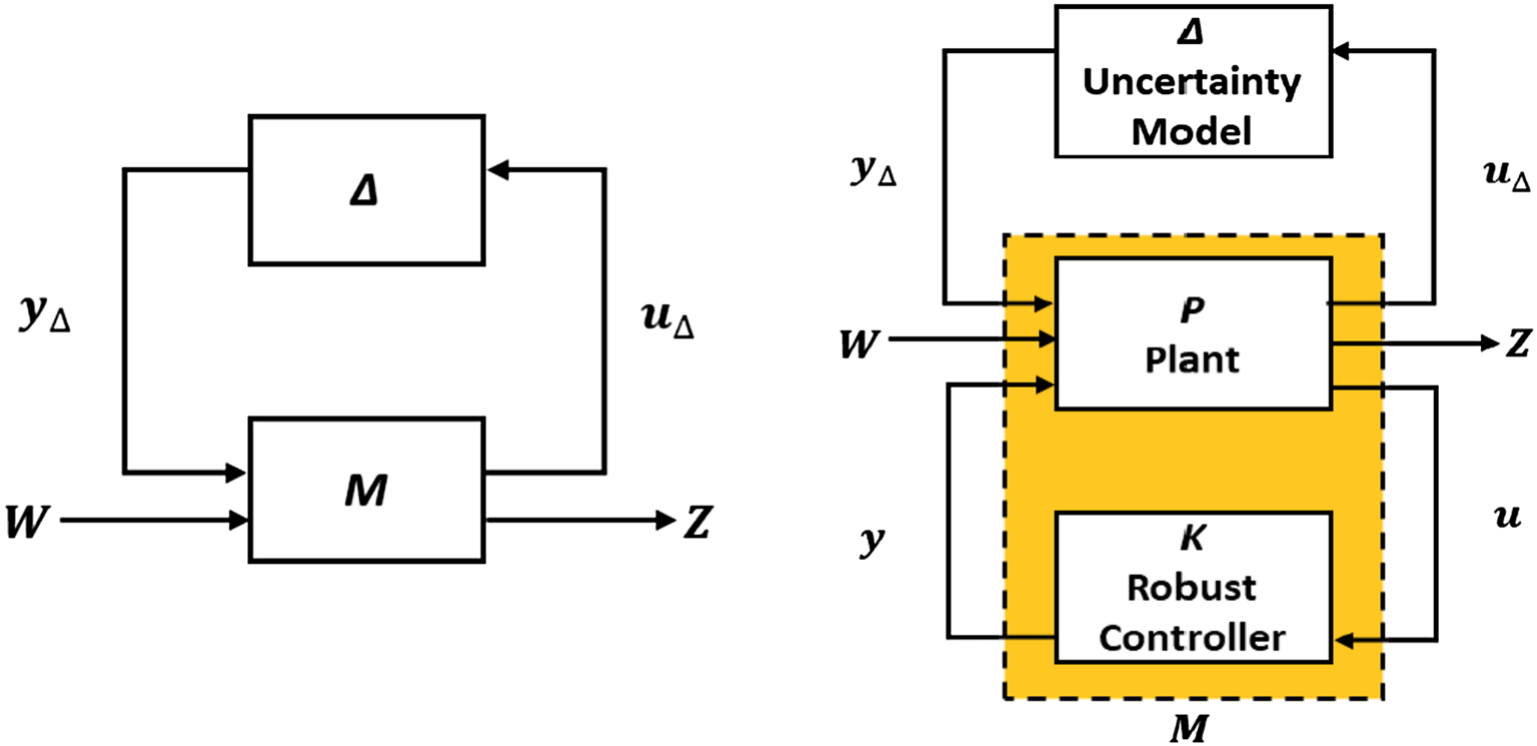
Structure of the µ-synthesis control system.

Figure 5 shows the structure of the µ-synthesis control system. Figure 5 (left) displays the normalized M-∆ structure with the plant and the uncertainty model ∆, whereas Fig. 5 (right) displays the closed loop P-∆-K structure with the plant, controller, and uncertainty model ∆. Where Z, W, y, ${u}_{\Delta }$, u, and ${y}_{\Delta }$ are the control performance signals, exogenous inputs, control signals, measured input , outputs, and output perturbation signals of the uncertain block, respectively^32^.

The function of the µ-synthesis is defined in Eq. (7) where ∆ represents the structure and unstructured uncertainties and is defined in Eq. (8).

7 $${\mu }_{\Delta }(M)= {\frac{{1}}{{{{\min\limits_{\Delta \in \Delta}}}\{{\sigma }(\Delta ) : |I-M\Delta |=0\}^{\prime}}}}$$

8 $$\Delta =\{diag \left[{\delta }_{1}{I}_{r1},\dots \dots ..,{\delta }_{k}{I}_{rk},{\Delta }_{1}\dots \dots ,{\Delta }_{f}\right],{\delta }_{i}\epsilon C, {\Delta }_{j} \epsilon {C}^{{k}_{i}x{k}_{j}}\}$$

**Figure 6:**
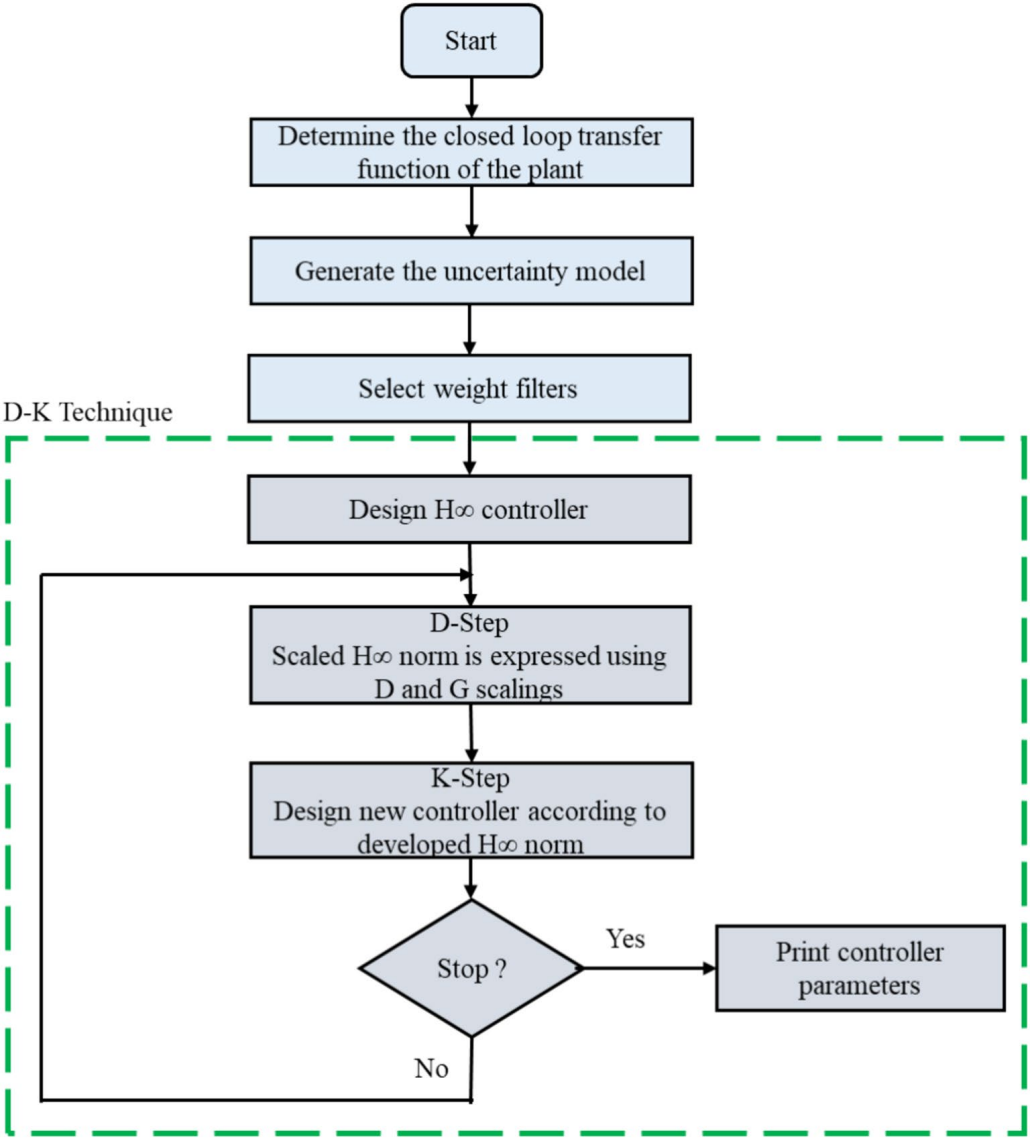
Flowchart of the proposed µ-synthesis control technique.

D–K iteration method^30^ is used to find the µ-synthesis controller parameters. This procedure resolves a series of scaled H∞ issues. The D and G scalings are used to express a scaled H∞ norm and are characterized by frequency dependence and utilizing the uncertainty structure. The following steps can be used to summarize the D–K iteration solving process^29^. The first step is to find the H∞ controller that minimizes the plant closed loop gain. In the second step, a scaled H∞ norm is expressed using D and G scalings (called the D step). A new controller is designed in the third step according to the H∞ norm in the second step. The forth step is the stop criterion. In this step the second and third steps are repeated until the stopping criterion which depends on whether the robust performance is met. Equation (9) indicates the general formula of the output continuous-time controllers using the µ-synthesis technique. Figure 6 shows the flowchart of the proposed control technique indicating the D-K solving steps.

9 $${C}_{\upmu }\left(S\right)= \frac{{a}_{1}\ast {s}^{2}+{b}_{1}\ast s+{c}_{1}}{a\ast {s}^{2}+b\ast s+c}$$

where $a, b, c, {a}_{1},{ b}_{1}, and {c}_{1}$ are the µ-synthesis controller parameters indicated in Appendix 1 in supplementary file. ${C}_{\upmu }\left(S\right)$ is the transfer function of the sub-system µ-synthesis controller.

Robust performance and robust stability tests should be performed after designing the µ-synthesis controller. The maximum value of µ for robust performance should satisfy the performance criterion ${\Vert \Delta \Vert }_{\infty }$ < 1 to guarantee robust performance; additionally, the upper bound of µ in the worst case gain should satisfy the same relation to grantee robust stability for the studied system.

For a certain design problem, choosing weighting filters is a challenging process that requires fine tuning and ad hoc decisions. Giving a generic weighted filter formula that will work in all scenarios is very difficult. In ^32–39^, the general requirements for the choosing of weighting filters are discussed, which may be helpful in selecting an initial weight.

Performance weight filters are applied to the control actions to keep the control sensitivity at a low limit and operate each sub-system in a specific frequency band. The weight parameter of ${WZ}_{1}$ shown in Fig. 2 is selected to minimize the cost function in Eq. (10).

10 $${\Vert \left[\begin{array}{c}S\ast {W}_{lp}\\ T\ast  {W}_{hp}\end{array}\right]\Vert }_{2}$$

where *W*_*lp*_ is a low pass filter for the sensitivity function and *W*_ℎ*p*_ is a high pass filter for complementary sensitivity. Where *T* and *S* are the complementary sensitivity and sensitivity of the closed loop system, respectively. For optimal reference tracking, the sensitivity function (*S*) should have low gain at low frequencies and high gain at high frequencies to avoid overshoot; therefore to weight the sensitivity function, a low pass filter *W*_*lp*_ should be chosen^38^. On the other hand, *T* should be near zero for noise suppression and near unity for successful reference tracking. A high pass filter is used to weight the complementary sensitivity function^30^ since the majority of noise energy is focused on high frequencies and the reference input signal occurs at low frequencies. Equation (11) can be used to obtain the weighting functions of *S* and *T*.

11 $$ WZ\left( S \right) = \frac{{s{\text{/}}M_{P}  + W_{C} }}{{s + \varepsilon \ast W_{C} }} $$

where ${W}_{C}$ is the minimum bandwidth frequency, ${M}_{P}$ is the maximum peak magnitude, and $\varepsilon$ is the maximum tracking error. The crossover frequency of the sensitivity weighting function should be selected to minimize the maximum desired closed-loop time constant. Since noise was not taken into account in this work, *W*_ℎ*p*_ is zero.

**Figure 7:**
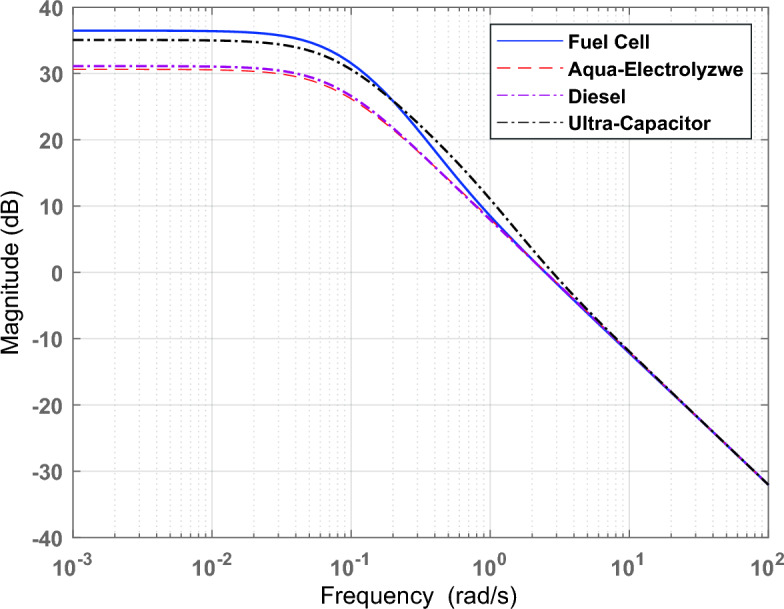
Bode diagrams for sub-systems.

The weight functions ($${WZ}_{2}$$, $${WZ}_{3}, {WZ}_{4}, and {WZ}_{5})$$ are selected to operate each sub-system in a specific frequency band, such as operating ultra-capacitor in the high frequency band and operating diesel generator, fuel cell, and aqua-electrolyzer in the low frequency band due to their mechanical nature. Figure 7 shows the Bode diagram for each sub-system.

**Figure 8:**
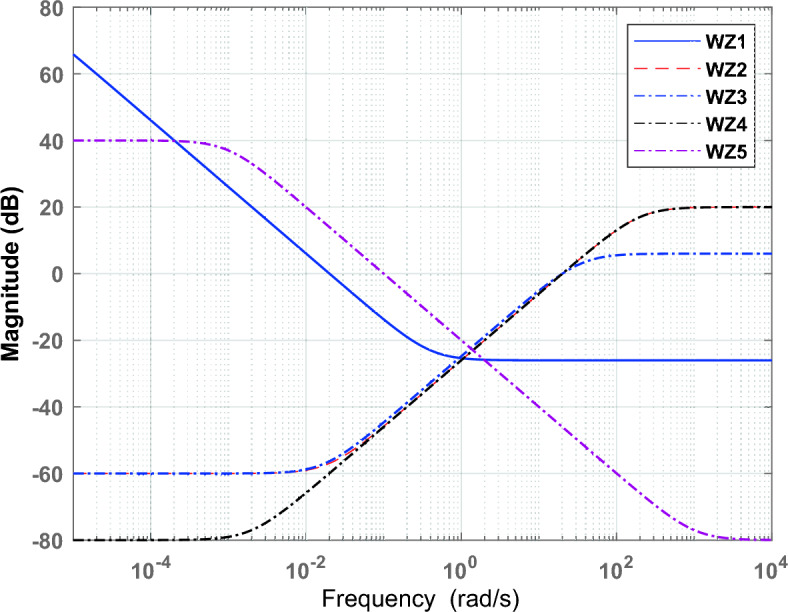
Bode diagrams for weighting filters.

The selection of weighting filters ($${WZ}_{1}$$, $${WZ}_{2}$$, $${WZ}_{3}, {WZ}_{4}, and {WZ}_{5})$$ is given in Eq. (12) and their Bode diagrams are shown in Fig. 8.

12 $${WZ}_{1}=\frac{0.05 s + 0.02}{s + 2e-6},{WZ}_{2}=\frac{10 s + 0.199}{s + 199},{WZ}_{3}=\frac{2 s + 0.03464}{s + 34.64},{WZ}_{4}=\frac{2 s + 0.0199}{s + 199},{WZ}_{5}=\frac{0.0001 s + 0.1}{s + 0.001}$$

### CHIO-PID and CHIO-FOPID controllers

The concept of the CHIO was developed based on the herd immunity theory to combat the coronavirus (COVID-19) pandemic. The contact of infected individuals with other members determines the coronavirus infection spreading rate. Health experts recommend social distancing as a way to shield other members from the disease. Herd immunity is a state reached by a population when the majority of the population is immune, resulting in a decrease in disease spread.

**Figure 9:**
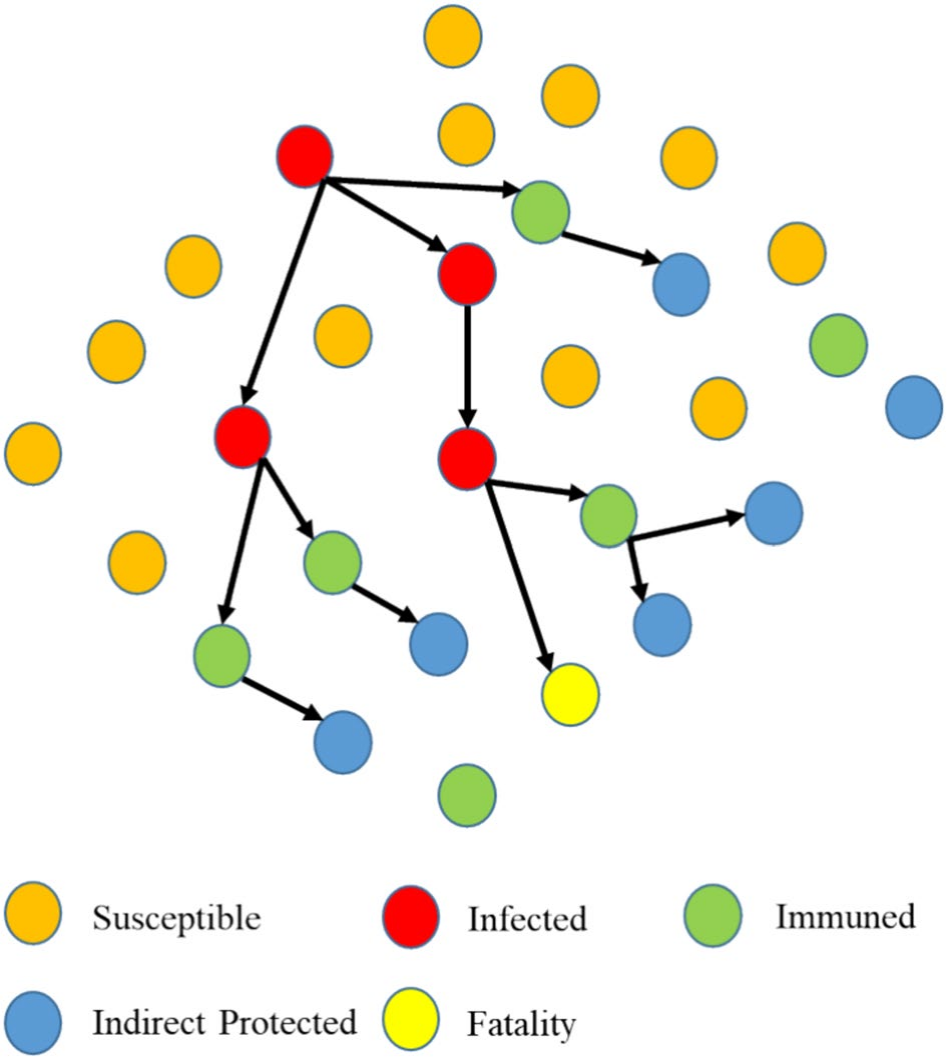
Types of individuals.

The previously illustrated concepts are modeled by an optimization algorithm. CHIO simulates both the herd immunity approach and the principles of social distancing. The herd immunity individual cases are classified into three categories: susceptible, infected, and immuned. This approach is used to determine how the newly generated solutions update their genes according to social distancing strategies. Figure 9 indicates the three types of individuals.

**Figure 10:**
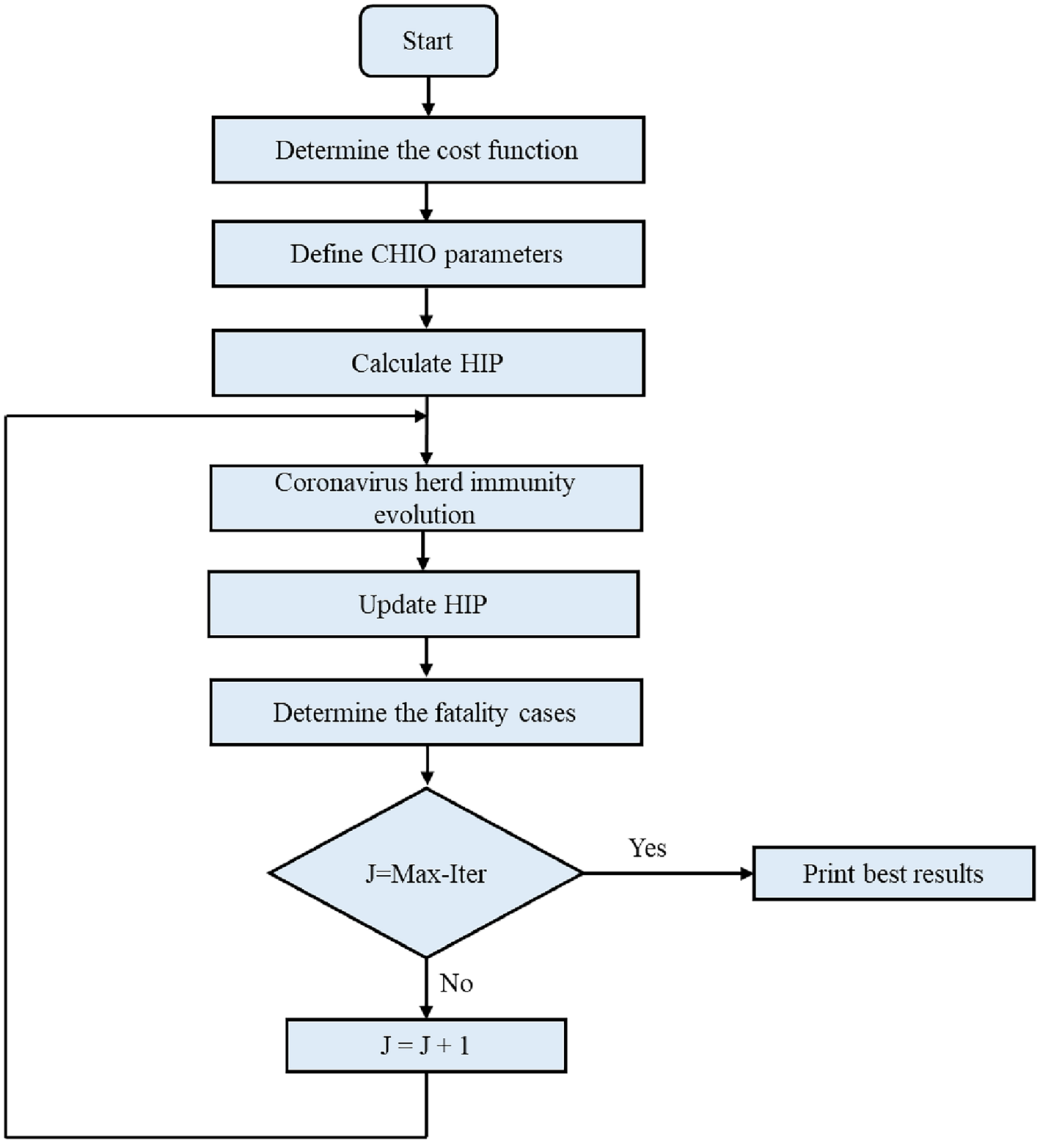
The CHIO-PID and CHIO-FOPID flowchart.

CHIO-PID and CHIO-FOPID controllers are designed for each sub-system for comparison purposes with the µ-synthesis controller. Figure 10 shows the design steps of the CHIO-PID and CHIO-FOPID controllers^41^. The steps are summarized as follow: the first step is the determination of the cost function; in this case, the cost function minimizes the integral absolute time error as a function of the sub-system controller’s parameters.

The CHIO parameters are defined in the second step; the parameters are defined as follow ${x}_{i}$ is the decision variable with range of ${x}_{i}\in [{l}_{i},{u}_{i}]$ where ${l}_{i}$ and ${u}_{i}$ refer to the lower and upper limits of ${x}_{i}$,$n$ is the number of variables, Max-Iter is the maximum number of iterations, population size (HIS), maximum age, and ${C}_{o}$ is the number of infected cases.

In the third step, the herd immunity population (HIP) is calculated according to Eqs. (13) and (14).

13 $$HIP=\left[\begin{array}{cccc}{x}_{1}^{1}& {x}_{2}^{1}& \dots & {x}_{n}^{1}\\ {x}_{1}^{2}& {x}_{2}^{2}& \dots & {x}_{2}^{2}\\ \vdots & \vdots & \dots & \vdots \\ {x}_{1}^{HIS}& {x}_{2}^{HIS}& \dots & {x}_{2}^{HIS}\end{array}\right]$$

14 $${x}_{i}^{j}= {l}_{i}+\left({u}_{i}- {l}_{i}\right) x U(\text{0,1})$$

In the fourth step, calculate ${x}_{i}^{j}\left(t+1\right)$, which is affected by social distancing as described in^36^. HIP is updated in the fifth step according to the fourth step and the updated values satisfy the cost function. The fatality cases are determined in the sixth step, where the cost function of ${x}_{i}^{j}\left(t+1\right)$ does not improve with increasing age. In the last step the stopping criterion is achieved until the maximum number of iterations is reached. Equations (15) and (16) indicate the general formula of the output controllers using CHIO-PID and CHIO-FOPID controllers respectively.

15 $${C}_{PID}\left(S\right)= {K}_{P}+ {K}_{I} / s +{K}_{D} \ast s$$

16 $${C}_{FOPID}\left(S\right)= {K}_{PP}+ {K}_{II} / {s}^{\alpha } +{K}_{DD} \ast {s}^{\beta }$$

where ${K}_{P},{K}_{I}, and {K}_{D}$ are the PID controller parameters indicated in Appendix 2 in supplementary file. ${C}_{PID}\left(S\right)$ is the transfer function of the sub-system PID controller. ${K}_{PP},{K}_{II}, and {K}_{DD}$ are the FOPID controller parameters indicated in Appendix 3 in supplementary file. ${C}_{FOPID}\left(S\right)$ is the transfer function of the sub-system FOPID controller.

## Simulation results

A decentralized µ-synthesis controller for each sub-system is designed based on the uncertainty model and weight actions according “Uncertainty modeling” and “Proposed controllers” and comparisons between the µ-synthesis controller, CHIO-PID, and CHIO-FOPID controllers are performed. MATLAB® R2020b was used to simulate the system using the proposed controllers.

**Figure 11:**
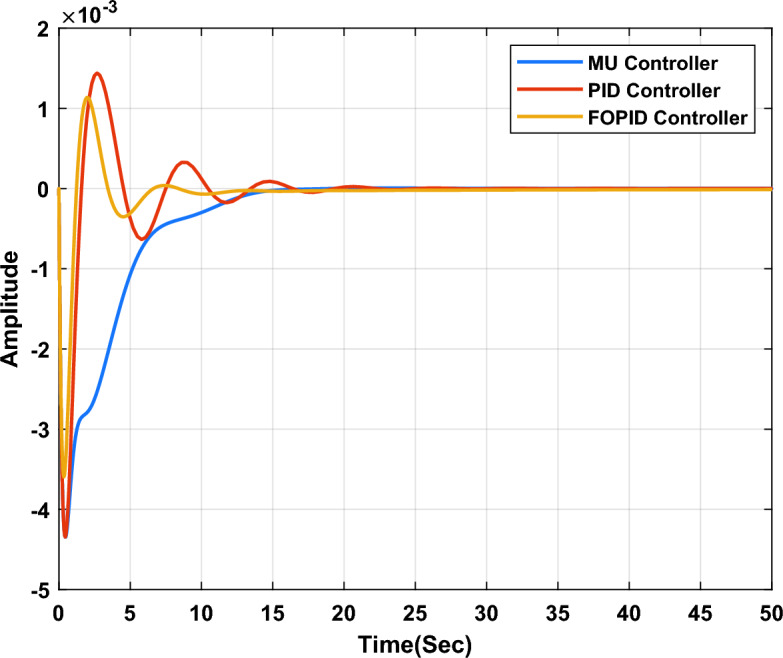
Frequency deviation of the microgrid for the µ-synthesis, PID, and FOPID controllers.

**Figure 12:**
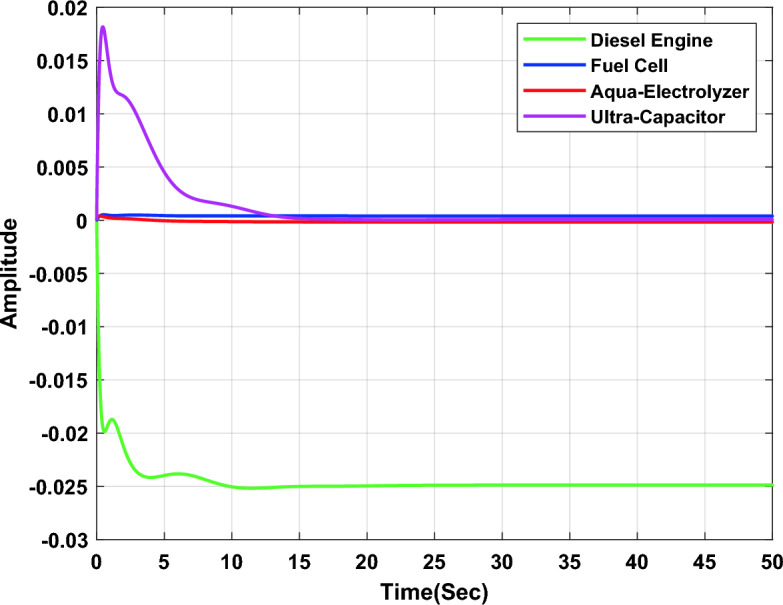
Control actions for µ-synthesis controllers.

**Figure 13:**
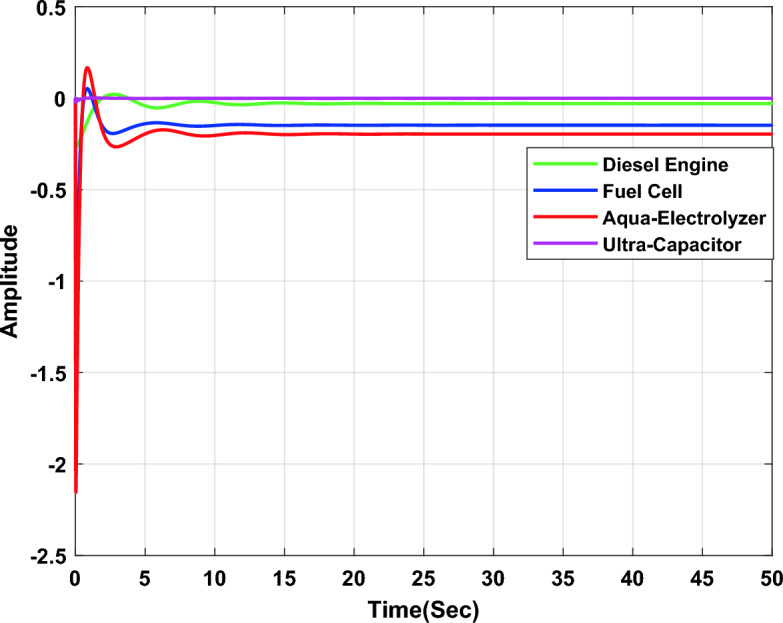
Control actions for CHIO-PID controllers.

**Figure 14:**
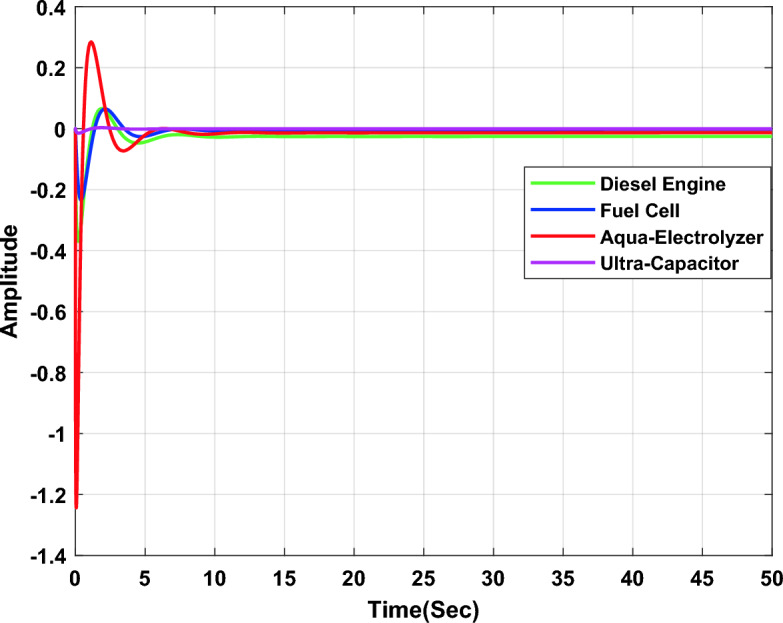
Control actions for CHIO-FOPID controllers.

In the first study case, a µ-synthesis controller is compared with CHIO-PID and CHIO-FOPID controllers at a load change of 10% and an uncertainty level of 40%. The frequency deviation of the microgrid for all controllers is compared in Fig. 11, which indicates that, the µ-synthesis controller has a better dynamic response with a settling time of 13.02 s compared with 35 s and 30.56 s for the PID and FOPID respectively. The controller’s actions are shown in Figs. 12, 13, and 14, which indicate that, the controller’s actions of the µ-synthesis controller are within acceptable limits compared with PID and FOPID.

**Figure 15:**
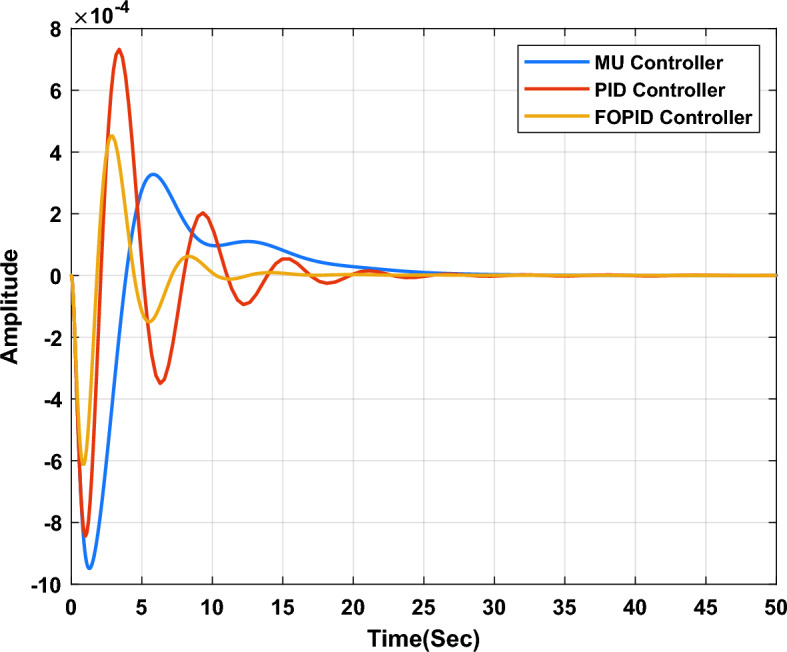
Frequency deviation of the microgrid for the µ-synthesis, PID, and FOPID controllers.

**Figure 16:**
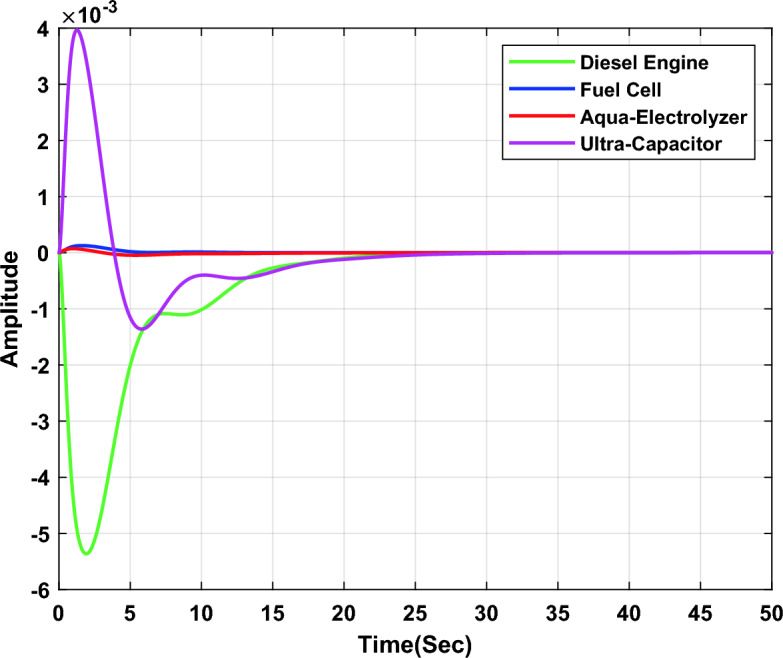
Control actions for µ-synthesis controllers.

**Figure 17:**
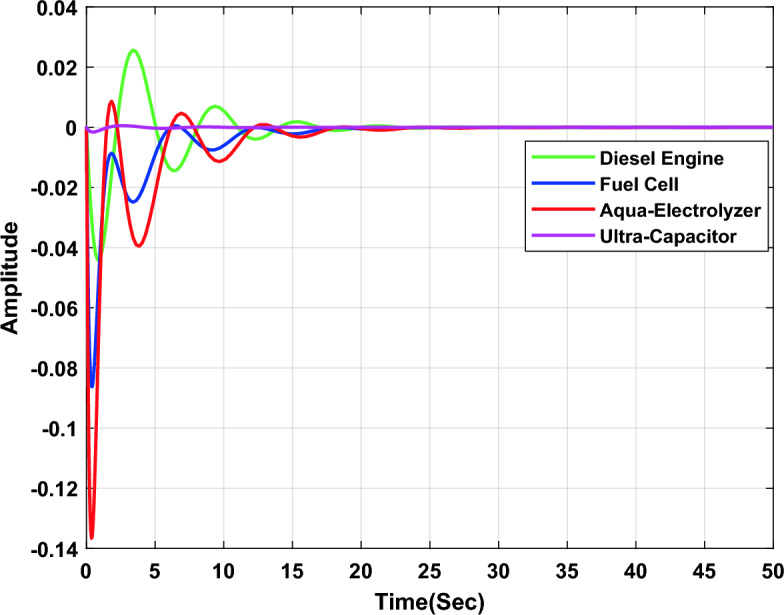
Control actions for PID controllers.

**Figure 18:**
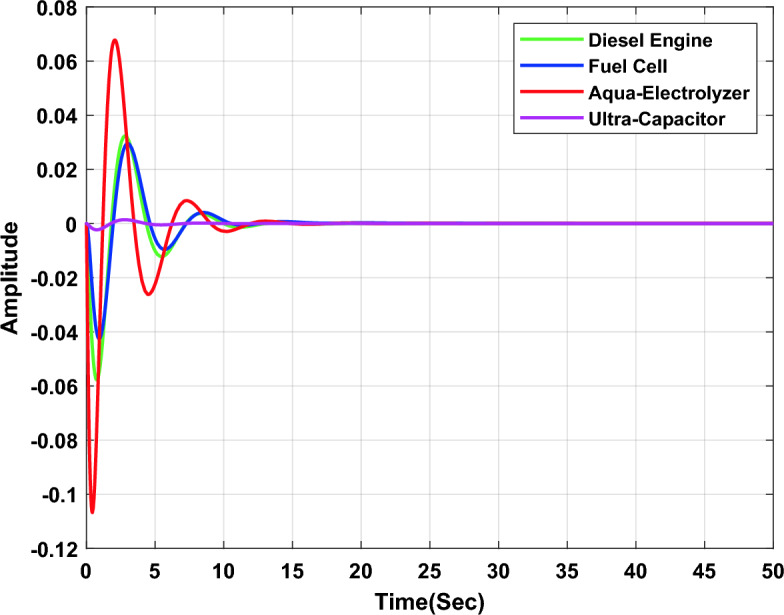
Control actions for FOPID controllers.

In the second study case, a µ-synthesis controller is compared with a CHIO-PID and CHIO-FOPID controllers at wind power change of 10% and an uncertainty level of 40%. The frequency deviation of the microgrid for all controllers is compared in Fig. 15, which indicates that, µ-synthesis controller has a better dynamic response with a settling time of 22.22 s compared with 46.48 s and 31.36 s for the PID and FOPID respectively. The controller’s actions are shown in Figs. 16, 17, and 18, which indicate that, the controller’s actions of the µ-synthesis controller are within acceptable limits compared with PID and FOPID.

Table 3 summarizes the step response specifications for the microgrid frequency with all types of controllers in each study case.

**Table 3:** Step response specifications.

Specifications	10% Load change			10% Wind power change		
	µ-synthesis	PID	FOPID	µ-synthesis	PID	FOPID
Steady state error	3.93e−9	93e−8	1.3e−5	2.45e−9	3.2e−8	2.58e−7
Settling time (s)	13	35	30.56	22.2	36	31.36
Maximum overshoot	7.18e−6	0.0014	0.0011	3.27e−4	7.33e−4	4.5e−4
Rise time (s)	2.19e−6	0.013	1.12	5.14e−5	0.0596	0.002
Peak time (s)	0.46	16.34	15.91	1.26	14.5	11.61

Robust stability is checked using the (robstab) command in MATLAB® 2020b for all controllers. This command is a robust tool used to evaluate the stability of control systems in the presence of uncertainties. It begins by defining the system's transfer function and representing uncertainties within specified bounds. The command then formulates the robust stability problem using a Linear Fractional Transformation (LFT) representation of the uncertain system. It calculates the structured singular value (μ) across all frequencies. If μ remains less than 1 for all frequencies, the system is considered to be robustly stable. This process provides a rigorous and thorough assessment, ensuring that the controller can handle real-world uncertainties effectively^42,43^.

**Figure 19:**
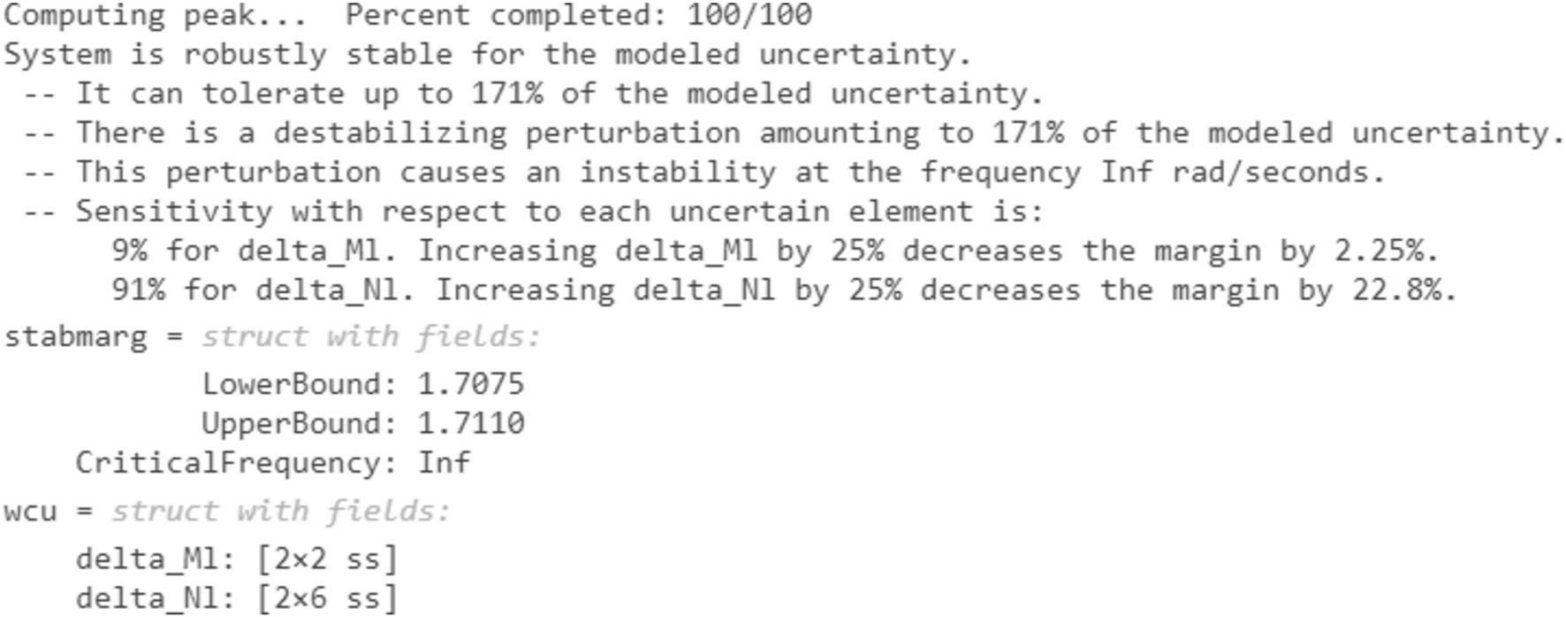
Robust stability test from MATLAB for µ-synthesis controller.

**Figure 20:**
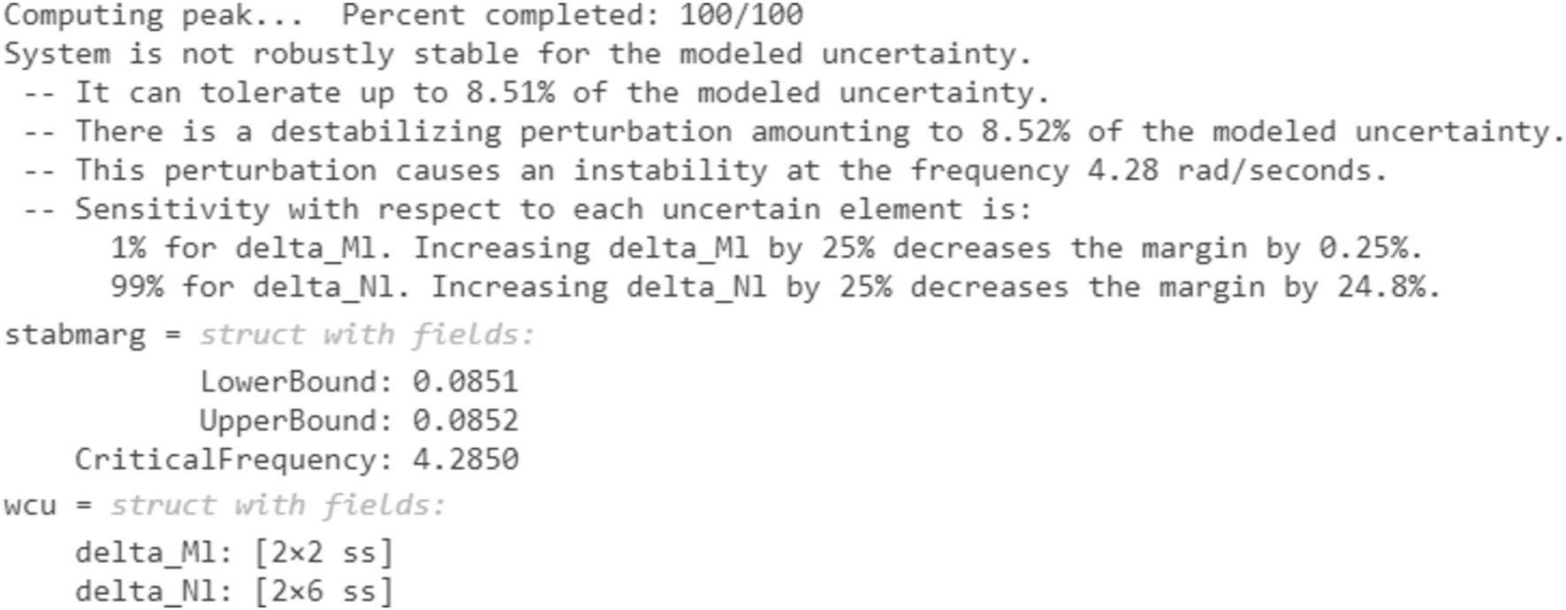
Robust stability test from MATLAB for CHIO-PID controller.

The reports generated from MATLAB® are indicated in Figs. 19 and 20. According to the generated reports, the µ-synthesis controller has robust stability against a high level of uncertainty. It can tolerate up to 171% of the modeled uncertainty compared with 8.52% for the CHIO-PID controller.

**Figure 21:**
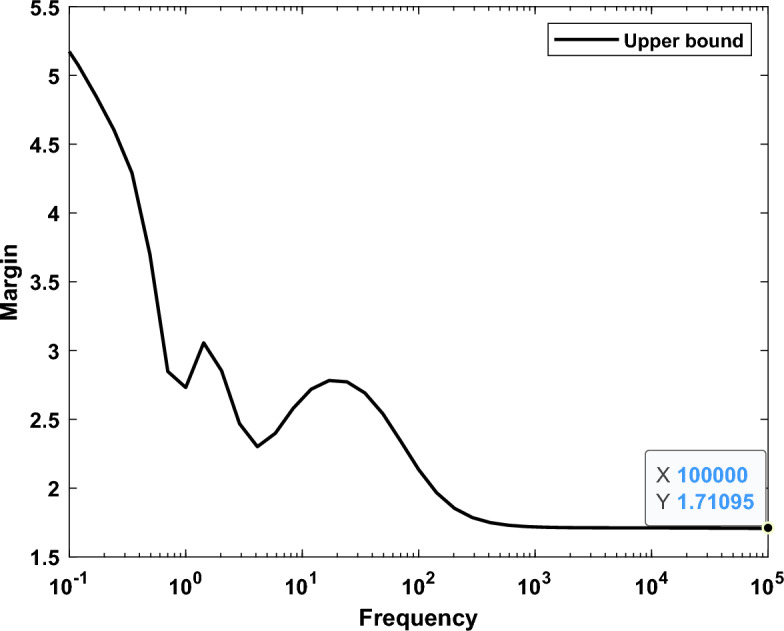
Frequency response according to the stability margin.

**Figure 22:**
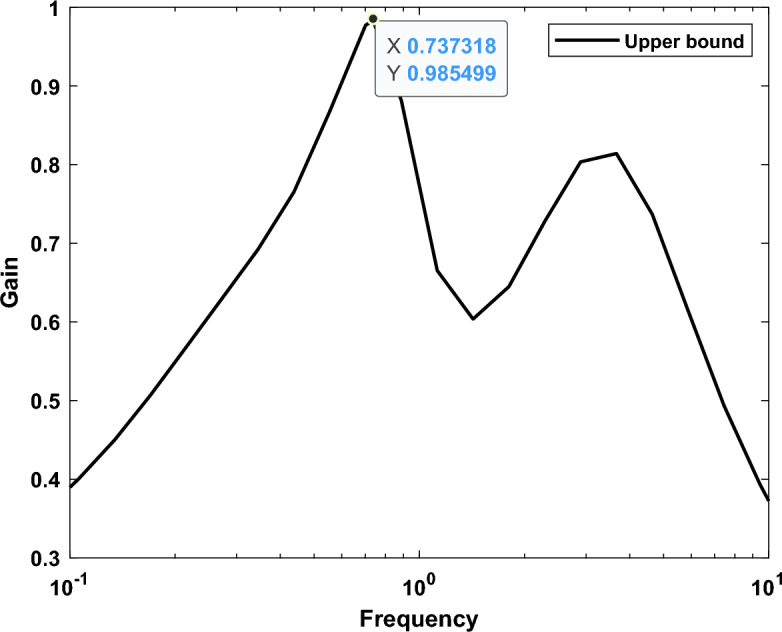
Frequency response according to the worst case gain.

The maximum value of µ synthesis for robustness performance is 0.58, which satisfies the performance criterion ${\Vert \Delta \Vert }_{\infty }$ < 1. Additionally, the worst case gain upper bound of μ is equal to 0.9847. Figures 21 and 22 show the frequency response according to the stability margin and the frequency response according to the worst case gain respectively.

## Conclusion

In this study, a precision frequency regulation approach is introduced for isolated microgrids utilizing continuous-time µ-synthesis control techniques. Specifically, decentralized fixed structure second-order µ-synthesis controllers were designed for each sub-system generation unit within the microgrid. Tailored performance weights were meticulously selected to uphold low control sensitivity and ensure precise operation within predefined frequency bands for each sub-system. Through comprehensive simulation results, the proposed µ-synthesis controller showcased its effectiveness in regulating microgrid frequency, demonstrating robust performance and stability under high levels of uncertainty.

Comparative analyses, pitting the controller against the CHIO-PID and CHIO-FOPID controllers, underscored its superior performance. The presented controller not only exhibits a low order for practical implementation but also proves its robustness in the face of significant uncertainty levels. Notably, under the most challenging and uncertain conditions studied, the controller remained stable, showcasing a worst case gain upper bound of μ equal to 0.9847. This signifies its robustness and reliability in ensuring microgrid stability. Additionally, a clear illustration of the design steps involved in implementing the proposed controller is provided, emphasizing its practicality and ease of implementation.

Overall, this study presents a compelling solution for precise frequency regulation in isolated microgrids, offering a robust and practical alternative in the presence of evolving energy landscapes. Investigating the integration of machine learning techniques for real-time optimization in microgrid frequency regulation presents a promising avenue for future research.

## Supplementary Information

Supplementary Information.

## Data Availability

The datasets used and generated during the current study are available from the corresponding author upon reasonable request.
