# Supplementary material for: Continuous-time robust frequency regulation in isolated microgrids with decentralized fixed structure μ-synthesis and comparative analysis with PID and FOPID controllers
Source: Sci Rep. 2024 Sep 5;14:20800. doi: 10.1038/s41598-024-70405-7 (PMC11379935; doi:10.1038/s41598-024-70405-7)
Supplement: Supplementary file 1 — Supplementary Information. [file 41598_2024_70405_MOESM1_ESM.docx]

APPENDIX 1

| Subsystem | Controller Parameters | | | | | |
| --- | --- | --- | --- | --- | --- | --- |
|  | $a$ | $b$ | $c$ | $a_{1}$ | $b_{1}$ | $c_{1}$ |
| Diesel Generator | 1 | 110.1 | 0.0002645 | 6.314 | 438.7 | 174.7 |
| Fuel Cell | 1 | 110.1 | 0.0002645 | -0.1173 | -12.13 | -2.808 |
| Aqua-Electrolyzer | 1 | 110.1 | 0.0002645 | -1.363 | -8.959 | 1.146 |
| Ultra-Capacitor | 1 | 110.1 | 0.0002645 | -8.516 | -459.9 | -0.4684 |

APPENDIX 2

| Subsystem | Controller Parameters | | |
| --- | --- | --- | --- |
|  | $K_{P}$ | $K_{I}$ | $K_{D}$ |
| Diesel Generator | 43 | 14 | 10 |
| Fuel Cell | 6 | 70 | 60 |
| Aqua-Electrolyzer | 3.4 | 93 | 97.5 |
| Ultra-Capacitor | 0.8 | 0.5 | 0.9 |

APPENDIX 3

| Subsystem | Controller Parameters | | | | |
| --- | --- | --- | --- | --- | --- |
|  | $K_{P}$ | $K_{I}$ | $K_{D}$ | $\alpha$ | $\beta$ |
| Diesel Generator | 92.5 | 49.7 | 20 | 0.8 | 0.7 |
| Fuel Cell | 33.3 | 38.18 | 0.3 | 0.2 | 0.9 |
| Aqua-Electrolyzer | 40 | 14.6 | 95.8 | 0.75 | 0.8 |
| Ultra-Capacitor | 1.5 | 9.5 | 1.34 | 0.5 | 0.5 |
